# Disseminated Cutaneous Histoplasmosis and Its Recurrence in an Apparently Immunocompetent Patient

**DOI:** 10.7759/cureus.60433

**Published:** 2024-05-16

**Authors:** Jorge Alberto Cortez-Vila, Carla Itzel Figueroa-Basurto, Rosa María Lacy-Niebla, Roberto Arenas, María Elisa Vega-Memije

**Affiliations:** 1 Department of Dermatology, Hospital General "Dr. Manuel Gea González", Mexico City, MEX

**Keywords:** lymphocitopenia, cutaneous, recurrence, itraconazole, immunocompetent, histoplasmosis

## Abstract

Histoplasmosis is a fungal infection caused by the fungus *Histoplasma capsulatum*. It can manifest in various ways, ranging from pulmonary to disseminated presentations. Most of the disseminated cases are seen in immunocompromised patients; here, we present an unusual case of an 81-year-old Mexican male with a history of cave exposure in his childhood, with 75 years of incubation period of the disease, who developed disseminated cutaneous histoplasmosis with no evident immunocompromising conditions. We considered the hypotheses of transient immunosuppression, CD4+ T lymphocytopenia, and immune senescence as the cause of this manifestation. The present case is also notable for its recurrence following therapy. This report underscores the challenges in diagnosing histoplasmosis in immunocompetent individuals and highlights the importance of long-term treatment and follow-up.

## Introduction

Histoplasmosis, also known as Darling’s disease, is a systemic mycosis caused by the fungus *Histoplasma capsulatum*. It predominates throughout America and Africa, where it is considered endemic. At the same time, there have been reports of cases in Oceania and Asia. In its various manifestations, it is found either localized in the lungs or disseminated to other organs. When presenting with cutaneous involvement, it is often caused by inhaling spores from the soil contaminated by birds and bats, which are then disseminated through the bloodstream [1,2].

Disseminated cutaneous primary histoplasmosis is a manifestation that rarely occurs without systemic involvement. There have been reports of disseminated histoplasmosis in healthy individuals and those with altered immune systems; however, it has been observed that among the latter, elderly men tend to be the most affected [3,4]. This means a strong index of suspicion is needed in these patients since the diagnosis of histoplasmosis is sometimes difficult in HIV-seronegative and immunocompetent individuals [5]. Direct inoculation may be suspected for a single lesion; however, this is difficult to prove if there is no mention of any traumatic history in the clinical record [6]. In this context, we present a particular case of cutaneous disseminated primary histoplasmosis in an immunocompetent patient without any other systemic involvement, who also experienced a relapse.

## Case presentation

**Figure 1:**
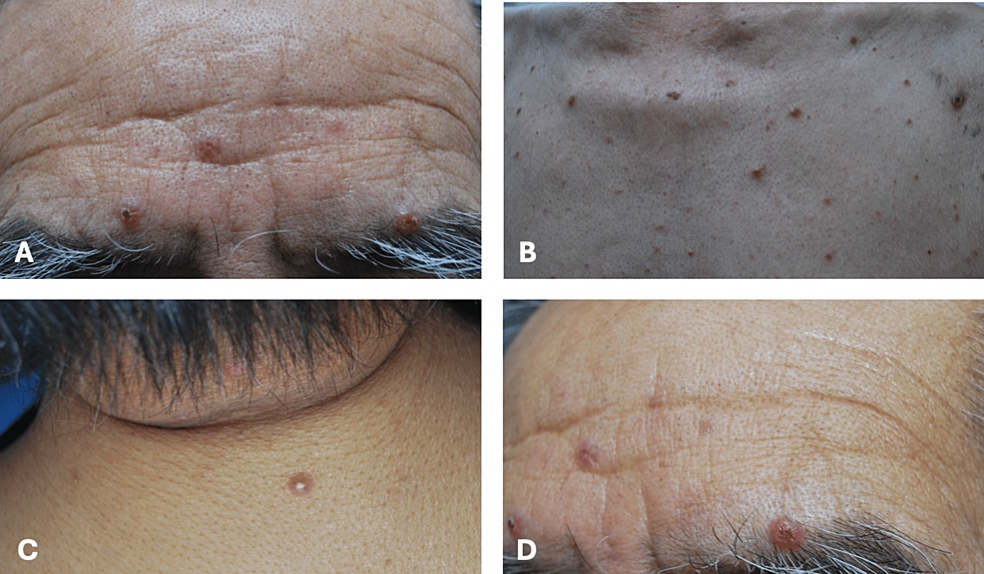
Cutaneous lesions as seen on the patient

An 81-year-old Mexican male patient presented with a disseminated bilateral (tending towards symmetry) dermatosis of three-month duration affecting the face, neck, anterior chest and upper back. It was characterized by multiple, isolated, 3- to 10-mm, erythematous, smooth, shiny, and some ulcerated nodules (Figures 1A-1D).

Previously, he was given oral doxycycline, oral non-steroid anti-inflammatory drugs, and over-the-counter ointments containing steroids, antibiotics and antifungal compounds. He recalled frequenting local caves in the Guanajuato state during his childhood, which made us suspect that the patient was a carrier of the fungus for 75 years. In terms of his medical history, he only reported systemic arterial hypertension treated with losartan.

**Figure 2:**
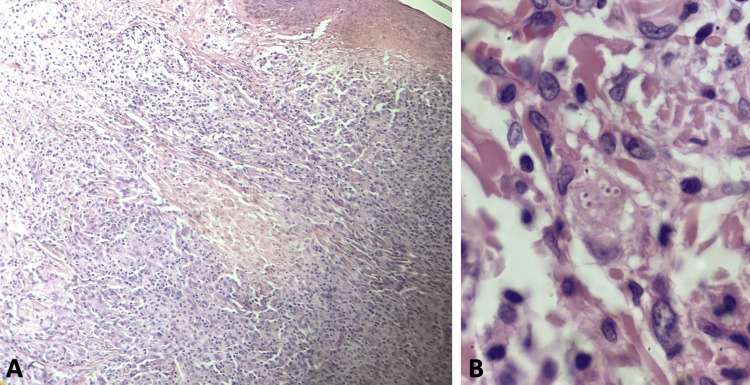
H&E stain results (A) A granulomatous-type infiltrate is observed in the superficial and middle dermis (H&E, 10x). (B) Small yeasts are observed (H&E, 100x).

**Figure 3:**
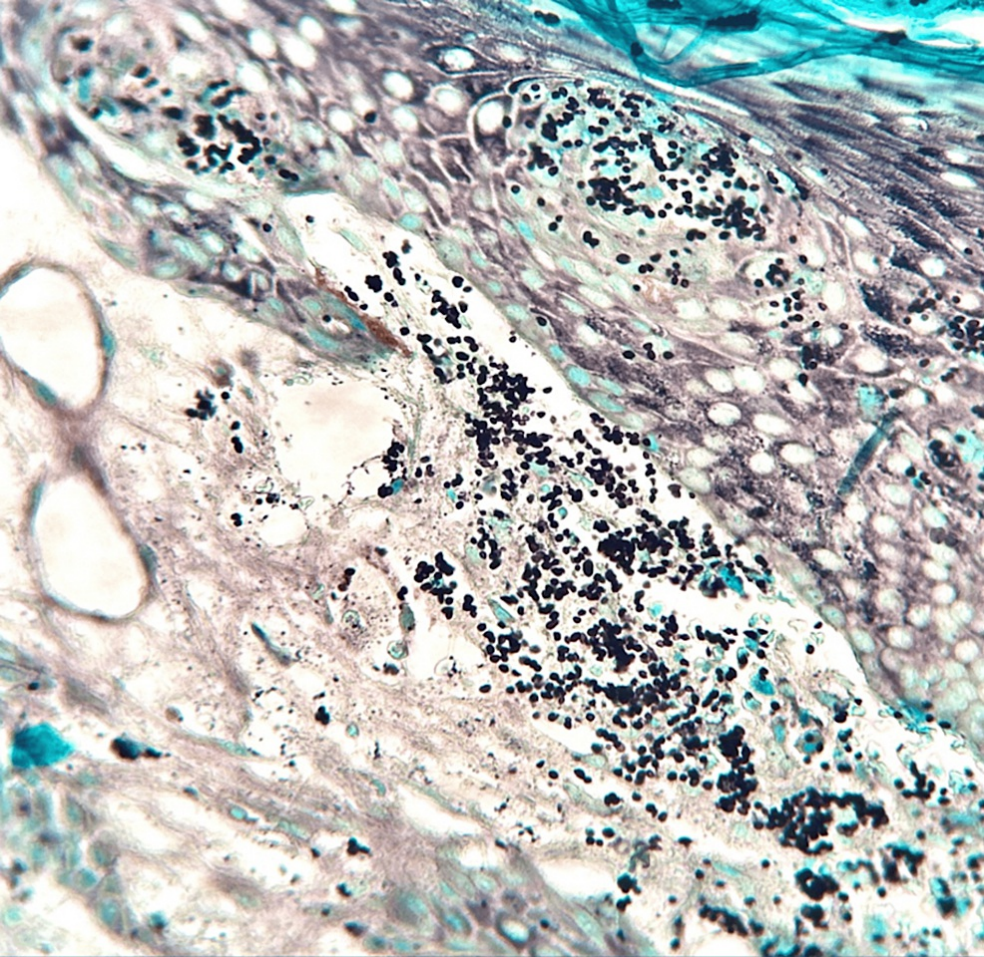
Histopathological features showing small spores surrounded by a transparent halo clustered inside the histiocytes (Grocott's stain, 40x)

The histopathological study of one of the nodules on the back revealed an inflammatory infiltrate composed of lymphocytes and histiocytes, some epithelioid cells, in a compact nodular arrangement, interspersed with some thick collagen fibers and small blood capillaries occupying the superficial and mid-dermis. Granulomas containing Langhans-type giant cells were also observed. H&E staining revealed in the superficial and middle dermis, a granulomatous-type infiltrate, as well as the presence of small yeasts (Figure 2). Grocott's stain highlighted the presence of small spores surrounded by a transparent halo, isolated and clustered inside the histiocytes (Figure 3).

**Figure 4:**
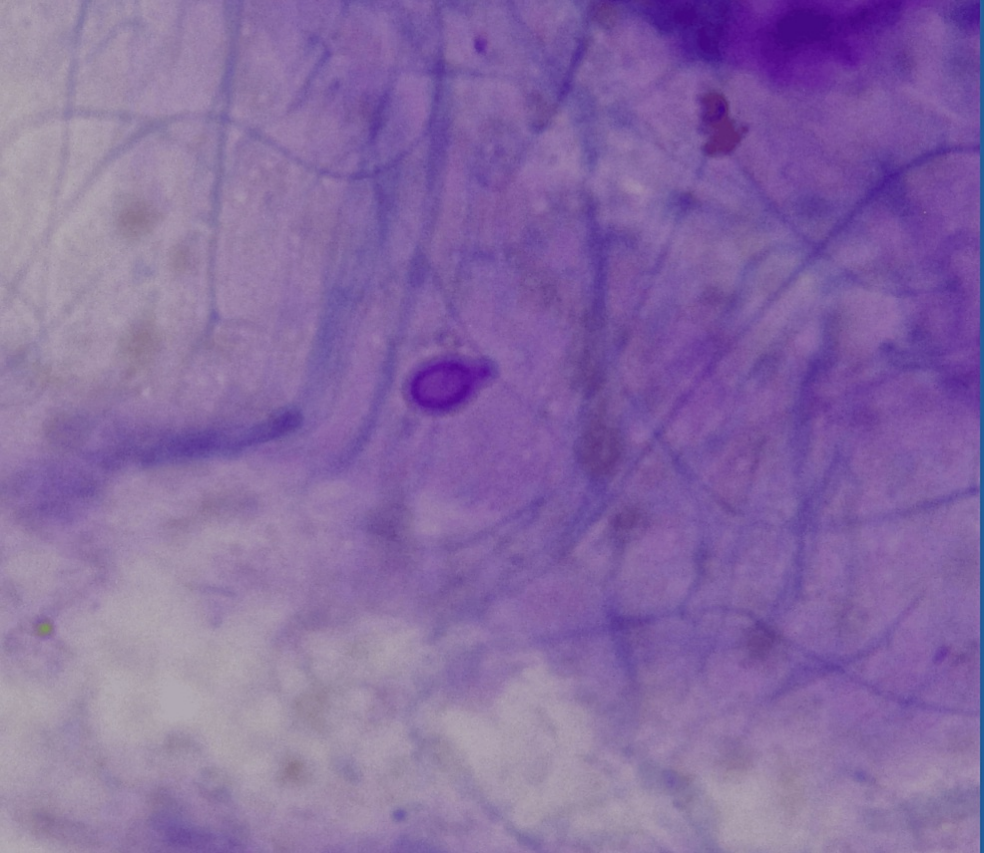
Budding yeast in the Gram stain smear

**Figure 5:**
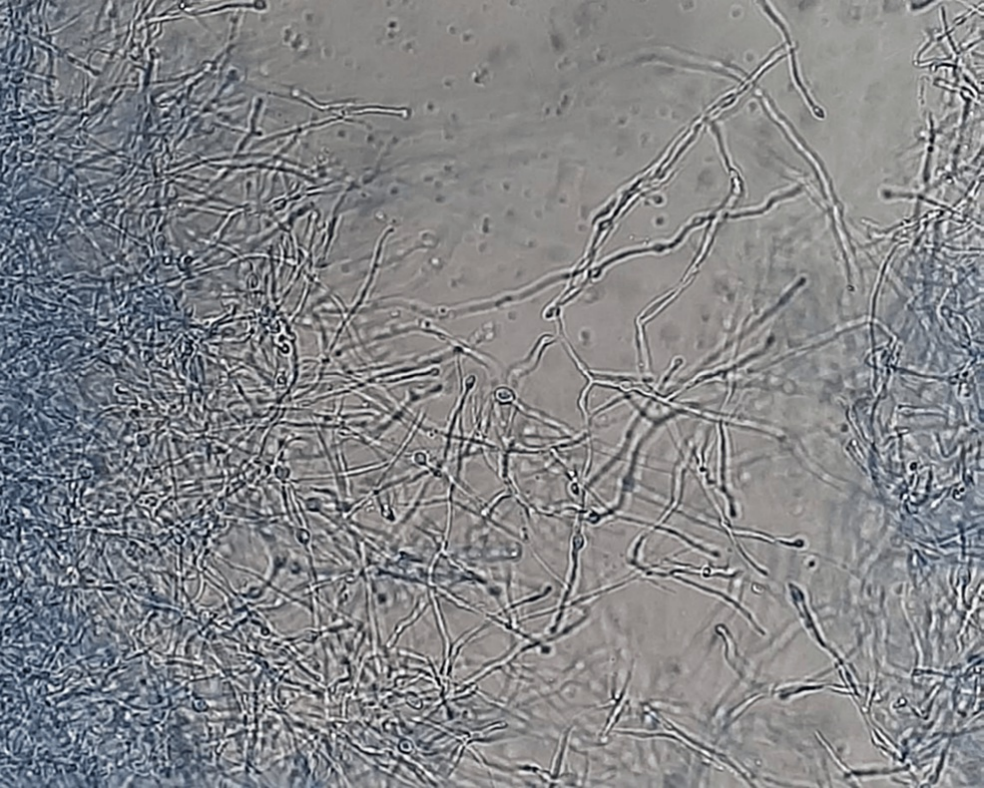
Microscopic examination of a culture, showing smooth-walled pyriform conidia and septate, branching filaments

Budding yeast was identified in the Gram stain smear (Figure 4), and the microscopic examination of a culture revealed the presence of branched and septate filaments, as well as smooth-walled pyriform conidia of *H. capsulatum *(Figure 5).

Computed tomography of the chest revealed no lung abnormalities. Clinical or laboratory data was not suggestive of any innate or acquired immunodeficiency. However, the complete blood count revealed a decline in the lymphocyte count, with an absolute value of 200/μL (normal, 1000-4800/μL). Itraconazole 200 mg/day was administered for five months, resulting in remission of the lesions. At the four-month mark, a new complete blood count showed lymphocyte levels within the normal range. Unfortunately, the patient was lost to follow-up and the treatment period was not completed.

**Figure 6:**
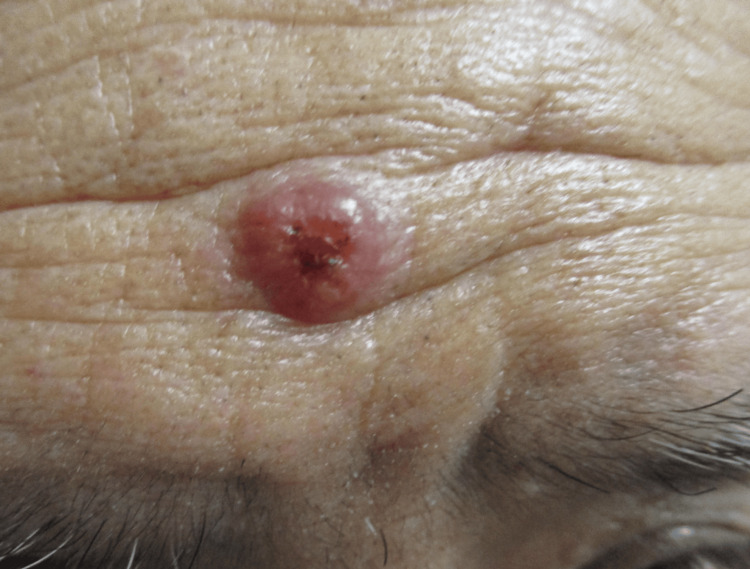
Recurrence of an ulcerated nodule on the frontal region of the face

A recurrence with a solitary 10-mm erythematous, smooth, shiny, and ulcerated nodule on the frontal region of the face (Figure 6) occurred a year and four months later. A Gram stain smear revealed the presence of *H. capsulatum*. Treatment with itraconazole 200 mg/day was restarted, resulting in remission after five months. The patient is now under follow-up, and in the sixth month of itraconazole treatment, which is prescribed for a total duration of one year. Also, he has been advised to continue follow-ups for more than a year due to the possibility of recurrence.

## Discussion

Systemic dissemination of histoplasmosis is due to a failure in the cellular immune response and is mainly observed in patients with HIV, being one of the AIDS-defining illnesses [5]. Other risk factors include kidney transplants, leukemias/lymphomas, vascular collagen disorders, and diabetes. Approximately 20% of patients have no identifiable risk factor [7]. Around 90% of immunocompetent hosts exposed to histoplasma present with subclinical symptoms or remain asymptomatic [3]. Therefore, cutaneous histoplasmosis in HIV-seronegative patients without an immunological system compromise requires a high threshold of suspicion [5]. In Latin America, *H. capsulatum *has been observed to have a certain affinity for the skin and can present as various elementary lesions, ranging from macules or papules to erythema nodosum or cellulitis [8]. Therefore, the fungus endemic geographic zone is one of the indicators that can lead to suspicion.

In Mexico, Histoplasma spp. spores are most commonly found in caves, mines, tunnels, and abandoned buildings containing bat guano [9]. It is estimated that 112 to 325 cases of histoplasmosis are annually reported, mainly in states with a large number of mines and caves, such as Guanajuato, Guerrero, Michoacán, Querétaro, Hidalgo, Chiapas, Yucatán, among others [10]. These data could provide clues for the diagnosis in this patient and also explain one of the likely routes of infection acquisition.

One of the proposed hypotheses in this case is that the condition resulted from the reactivation of an inactive focus due to transient immunosuppression [11], since the patient had a history of visiting mines in Guanajuato during childhood. However, it is difficult to conclude this because the patient did not present any risk factors that could alter his cellular immune response. Nevertheless, there is a possibility that we were dealing with a case of idiopathic CD4+ T lymphocytopenia, as there are several reports of patients presenting with histoplasmosis as the initial manifestation. Although this was not demonstrable in our patient, it is noteworthy that he presented with lymphocytopenia at the onset of the condition [12,13].

Idiopathic CD4+ T lymphocytopenia is a rare condition, defined by a CD4+ T lymphocyte count of less than 300/mm^3^ (500-1500 cells/mm^3^) or less than 20% of total lymphocytes for at least six weeks, without evidence of acquired or primary immunodeficiencies. This reduction in CD4+ T cells predisposes individuals to opportunistic infections by viruses, encapsulated fungi, or mycobacteria, as well as to neoplasms [14]. In a study conducted in 2023 [15], 91 patients with this diagnosis were included, and the most frequent opportunistic infections were identified, which were those related to papillomavirus (27 patients) and cryptococcosis (22 patients). Additionally, the presence of disseminated histoplasmosis in two patients was highlighted. However, it is essential to recognize that within this condition, physiological factors such as extremes of age and circadian rhythms may reduce the count or activity of CD4+ T cells [16].

Other immunological defects worthy of consideration as potential causes of skin lesions in apparently healthy patients include specific mutations in genes regulating critical points in the cellular immune response, such as those encoding for interferon-gamma and interleukin-12 receptors [5].

This perspective underscores the importance of considering idiopathic CD4+ T lymphocytopenia and its potential contributing factors in patients without known risk factors or a history of immunosuppression.

An intriguing observation was made in a case of cutaneous histoplasmosis, where a patient initially presented with transient depletion of CD4+ T lymphocytes, with a count of 161 cells/μL [12]. Following antifungal treatment, the lymphocyte levels returned to normal. A similar phenomenon was noted in our patient, although specific CD4+ T lymphocyte counts were not available. While this parallel is noteworthy, it's crucial to recognize that transient lymphocyte depletion can have multifactorial etiologies, and attributing it solely to fungal infection may be oversimplifying the situation. Further research is needed to explore and understand the complex interplay of factors contributing to lymphocyte dynamics in the context of fungal infections.

The second hypothesis suggests that the patient may have omitted some relevant history regarding the possibility of visiting a site contaminated with bat or bird droppings and may have been inoculated with a large quantity of spores [17], either through cutaneous contact or inhalation. This hypothesis is supported by a retrospective study [18] that revealed that 80% of patients with histoplasma infection did not have an occupational risk of developing the disease; additionally, 38% of the patients lived in Mexico City, which is consistent with our case.

The present case also highlights the recurrence of lesions, a phenomenon that may be mitigated by extending antifungal treatment with itraconazole for at least 12 months, even in cases of apparent clinical remission. It has been reported that in immunocompetent patients and cases of dissemination without central nervous system involvement, a response rate of 80%-100% is reached with this treatment period [19]. In our case, the patient was lost to follow-up and treatment was discontinued after five months, leading to the recurrence of lesions. Therefore, it was necessary to emphasize the crucial importance of continuous follow-up for at least one year, due to the risk of disease recurrence.

In an attempt to better understand the recurrence, the possibility of microorganism resistance to itraconazole was explored as a plausible hypothesis. However, the search for related reports or studies did not yield results supporting this theory, and currently there is no drug combination to reduce the resistance and recurrence of the disease.

Within the limitations of this case, it is acknowledged that establishing conclusions about the cause of why an immunocompetent patient presents with such manifestations is challenging. However, possibilities explored include the potential for reactivation of an inactive focus due to transient immunosuppression, alterations in CD4+ T cell counts, abnormalities in genes responsible for cellular immune response, and senescence of the immune system due to age.

## Conclusions

This case provides valuable lessons about the challenges and complexities associated with primary cutaneous histoplasmosis in apparently immunocompetent patients. It emphasizes the importance of future research to understand the causes due to which seemingly healthy individuals, like our patient, can develop such clinical presentations. Additionally, it highlights the need for understanding geographical and epidemiological factors contributing to infection acquisition, as well as proper long-term follow-ups to ensure compliance with the treatment period.
